# Association between sedentary behavior and low back pain; A systematic review and meta-analysis

**DOI:** 10.34172/hpp.2021.50

**Published:** 2021-12-19

**Authors:** Sadegh Baradaran Mahdavi, Roya Riahi, Babak Vahdatpour, Roya Kelishadi

**Affiliations:** ^1^Department of Physical Medicine and Rehabilitation, School of Medicine, Student Research Committee, Isfahan University of Medical Sciences, Isfahan, Iran; ^2^Child Growth and Development Research Center, Research Institute for Primordial Prevention of Non-communicable Disease, Isfahan University of Medical Sciences, Isfahan, Iran; ^3^Department of Epidemiology and Biostatistics, School of Public Health, Isfahan University of Medical Sciences, Isfahan, Iran; ^4^Department of Physical Medicine and Rehabilitation, School of Medicine, Isfahan University of Medical Sciences, Isfahan, Iran

**Keywords:** Sedentary behavior, Screen time, Smoking, Obesity, Coffee, Low back pain

## Abstract

**Background:** Sedentariness is a substantial risk for many chronic diseases. We aimed to investigate the correlation of sedentary behavior and its indicators with low back pain (LBP) among adults and children.

**Methods:** Original articles published up to April 28, 2020, using PubMed, Embase, Web of Science and Scopus were evaluated. Odds ratio (OR, 95% CI) was considered the overall effect size for desired associations.

**Results:** We reviewed 49 English articles with analytical observational study design, of which, 27 studies with cross sectional/survey design were retained in the meta-analysis. Among adults, sedentary lifestyle was a considerable risk factor for LBP (OR=1.24, 1.02-1.5); prolonged sitting time (OR=1.42, 1.09-1.85) and driving time (OR=2.03, 1.22-3.36) were the significant risk factors. Sedentary behavior was associated with LBP in office workers (OR=1.23). Moreover, excess weight (OR=1.35, 1.14-1.59) and smoking (OR=1.28, 1.03-1.60) were associated with LBP. Among children, sedentary lifestyle was a remarkable risk factor for LBP (OR=1.41, 1.24- 1.60); prolonged TV watching (OR=1.23, 1.08-1.41) and computer/mobile using and console playing time (OR=1.63, 1.36-1.95) were significant risk factors for LBP. Consumption of coffee, however, has yield conflicting results to be considered as a risk factor. Moreover, the researches on the correlation between sedentariness and high-intensity LBP are scarce and inconclusive.

**Conclusion:** Sedentary behavior, whether in work or leisure time, associates with a moderate increase in the risk of LBP in adults, children and adolescents.

## Introduction

Low back pain (LBP) is a paramount public health concern contributing to self-perceived disability and a high economic burden worldwide.^1,2^ It is associated with quality of life,^3^ long-term sickness, and early retirement as well.^4^ It is estimated that about 80% of the population has experienced an episode of LBP in their lives.^5^ LBP is more common in females and those between 40-69 years. LBP prevalence increases with aging, and the LBP in childhood associates with the corresponding figure in adulthood.^6^ It is shown that musculoskeletal symptoms in the lower back are correlated with other body segments, including the neck, upper back, and shoulders.^7^

Sedentary behaviors, on the other hand, are defined as activities with low energy expenditure, performed in rest positions. Sedentary behavior is a predictor of metabolic risk independent of physical inactivity.^8^ The health concerns associated with sedentariness are not merely attributable to lack of movement, but also to other simulations such as leisure or work screen time activities, including computer and internet use, TV (television) watching, cell phone use, and playing videogames.^9^ Besides, sedentary behavior is related with all-cause mortality concomitant with overweight and obesity, diabetes, and cardiovascular diseases.^10,11^ The association of sedentary behavior with musculoskeletal conditions such as LBP has been widely investigated among the population of workers and non-workers.^12^

With respect to sitting time, the findings regarding the association between sitting periods and LBP are inconsistent. One study among 704 participants demonstrated no independent association of sitting time in work time or the whole day with LBP. In this study, the body mass index moderated the mentioned association.^13^ In addition, the previous reviews did not mention any association between sitting time itself and LBP in leisure time or at work.^14-16^ However, a mixture of whole-body vibration, awkward postures, and prolonged sitting increased the risk of LBP.^14^ On the other hand, a study among 136 teaching staff reported that physical inactivity was related to LBP; but tobacco use and level of alcohol intake did not have such association with LBP.^17^ In a research among 665 blue-collar workers, a longer duration of sitting periods at work was beneficial for LBP.^18^ The difference in study design, measurement methods, and participants (or occupational groups) with different sedentary tasks may contribute to inconsistency for the correlation of sitting time and LBP in previous research.^18^

Excessive consumption of coffee and cigarette smoking were associated with an elevated likelihood of recurring LBP among 609 Polish residents. In this study hyperlipidemia, type 2 diabetes, and hypertension, were significantly associated with an increased likelihood of chronic LBP as well.^19^ Furthermore, in a cross-sectional study among 1221 school adolescents, playing video games (≥2 hours/day) and watching television (≥12 hours/week) were proposed as independent risk factors of LBP.^20^

Given the controversies in different articles, in this study, we investigated the relationship of different indicators of sedentary behavior and inactivity (including sitting time, screen time, smoking, consumption of coffee, and excess weight) with LBP, whether in leisure time or work time. We aimed to synthesize the available data to quantify the abovementioned associations to address inconsistencies in previous research. A brief systematic review has been presented in the case of a lack of required data for meta-analysis. In addition, we addressed the mentioned association among children and adolescents with a particular focus.

## Methods

### Search strategy

We performed a comprehensive search through electronic databases, including PubMed, Embase, Web of Science and Scopus for records published up to April 28, 2020. Based on a PEO framework (Patient/Population/Problem, Exposure, and Outcome) for the eligibility of the research question, we combined the indicators or equivalents of sedentary behavior on the one hand and the equivalents of LBP, on the other hand, for building the search strings. To facilitate the process of screening, we refined the results via the following filters wherever those were available in the search engines: article, journals, English language, full text, human studies. Appendix 1 shows the search strings in the abovementioned databases.

### Study design

All the analytical observational studies (cross-sectional, case-control, or longitudinal designs) in which the association between sedentary behavior and LBP was investigated, were favorable to be contained in our review. We did not include the experimental studies in which the effects of behavior intervention or experiment on LBP were studied.

### Patient/population/problem 

Studies with individuals with a specific medical condition such as scoliosis or renal failure who may spend most of their time for sedentary activities were excluded. Age range and type of occupation were not considered as limiting factors, i.e., children and adults with sedentary behavior and LBP were considered to be the subjects of our review.

### Exposure

Factors contributing to sedentary behavior, including sitting time, screen time, smoking, consumption of coffee, and body mass index, were considered to be the individuals’ exposures.

### Outcome

The onset or recurrence of nonspecific or mechanical LBP measured via different methods was the desired outcome in our study. We excluded the studies in which sciatica or any kind of radicular pain was investigated.

### Eligibility criteria and study selection

**Figure 1:**
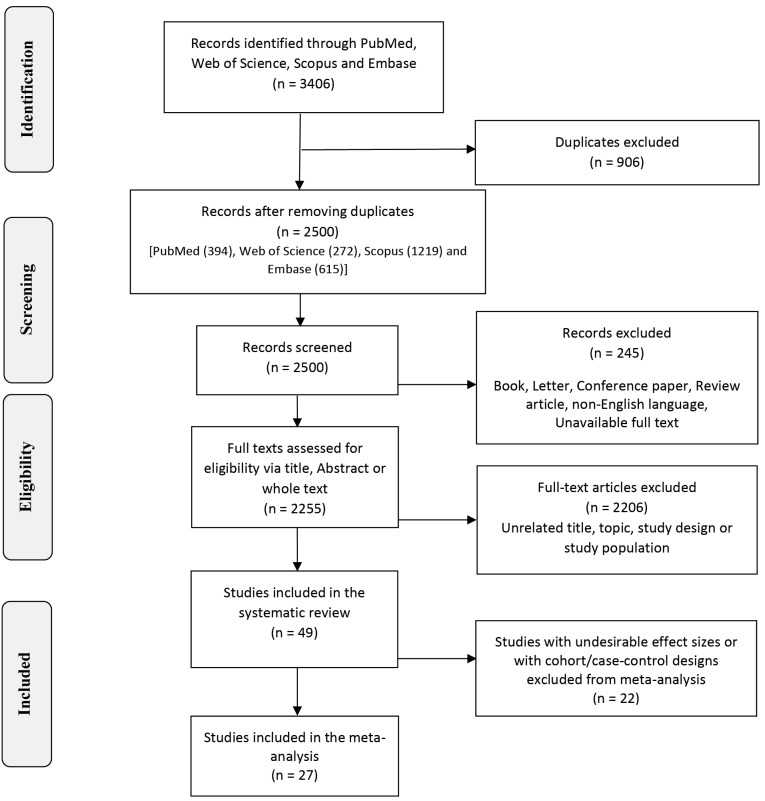
Flowchart of study selection for systematic review and meta-analysis.

Two independent reviewers (S.B.M and R.R) screened the relevant records using Endnote software (version 18) after removing duplicates. Thereafter, additional letters, books, review or conference papers, non-English language, and unavailable full texts that were not excluded in refining results in the search engines were excluded. Then, we reviewed the full texts of remained records entirely at the next step. We excluded the articles with topics, study design, or participants irrelevant to our review (Figure 1). Any disagreement was solved via a discussion for reaching consensus in the whole process.

### Assessment of study quality 

Two of the researchers (S.B.M and B.V) performed the study quality assessment independently. The STROBE scale, which consists of 22 items (combined version, 2007), was used for this purpose.^21^ One score to each item was given by each reviewer separately, if the criteria were fulfilled. For each paper, a mean STROBE scores ≥16.5, in the range 11 to 16.5 and lower than 11 were considered as high, moderate and low with respect to study quality, respectively.^22^ The agreement coefficient between researcher’s scores was more than 0.5.

### Statistical analysis

The desired effect size was considered as an odds ratio with 95% confidence interval (OR, 95% CI). Cochran’s Q and inconsistency index (I^2^) were used to explore the heterogeneity of the included articles. The random-effects model with DerSimonian and Liard method^23^ was used when data accumulated from studies, differed in ways that would have impacted on the results (e.g. subjects, exposure), otherwise the fixed effect model with inverse variance method was conducted. Then, the effect of each study on the pooled OR was assessed using sensitivity analysis. We performed subgroup analyses to evaluate the source of heterogeneity based on the following possible variables; type of sedentary behaviors and occupation type. The Begg’s and Egger’s tests were performed to explore publication bias. *P* value < 0.05 from both tests indicated remarkable publication bias. All analyses were conducted in the Stata, version 11.2 (STATA Corp, College Station, TX, USA).

### Review writing style

The items included in the current review have been written according to the checklist and flow diagram of the PRISMA version 2009.^24^

## Results

### Characteristics of included studies

Overall, 3406 records were recognized via a comprehensive search through biomedical sources. With excluding duplicates, books, letters, conference papers, review articles, non-English records, and unavailable full texts, 2255 records remained to be screened via title, abstract or whole text. Finally, we included 49 studies in this review, of which 27 were retained in the meta-analysis for different purposes,^19,20,25-49^ 18 were excluded due to undesirable effect sizes^1,5,13,50-64^ and 4 were excluded due to cohort/case-control designs.^65-68^ Figure 1 shows the process of study selection through a schematic flowchart.

With respect to study design of included articles in the review, 8 have been conducted in cohort/prospective design,^51,52,54,56,58,65-67^ 1 in case-control design,^55^ 1 in retrospective nested case-control design^68^ and the rest in cross-sectional/survey design. Among all studies, 15 were conducted in children or under graduated students.^20,26,29,31,35,38,40,46,47,51,53,55,61,62,66^ Only four studies investigated the association of coffee drinking with LBP.^19,32,57,62^ Complete information of these 49 articles has been presented in Table 1 and ordered chronologically from old studies to new ones.

**Table 1:** Summary of included studies in the systematic review

**First author;** **(year)** ^a^	**Study design; Study participant**	**Sample size**	**Age** **(years)**	**Sedentary behavior**	**Outcome**	**Assessment of low back pain**	**Main finding**	**Adjustment factors**	**Study quality** ^b^	**Ref.**
A. Burdorf (1993)^c^	Cross-sectional Sedentary worker in Rotterdam	275	41.5	Prolong sedentary posture belong work	LBP	Nordic musculoskeletal questionnaire	The adjusted risk for LBP among carne operator and straddle-carrier was significantly higher than office workers who spent lower sedentary daily work time.	Age, Physical activity, whole-body vibration, prolonged sitting, cold and draught in current work, working under severe pressure, and job satisfaction.	Moderate	50
T. Skov (1996)	Cross-sectional Danish salespeople	1306	39.3	Sedentary work (proportion of work time)	Chronic LBP	Self-reported Nordic questionnaire	Driving and sedentary work were related to neck and low back pain.	-	Moderate	25
R. Gunzburg (1999)^c^	Cohort Belgian students	392	9	Watching TV and playing video game (> 2 hours/day)	LBP	Questionnaire	More LBP in children who reported playing video games (> 2 hours/day).	-	High	51
P. R. Croft (1999)^c^	Cohort UK general population	4501	46.5	Watching TV more than 3h/day	Overall LBP	Questionnaire	There is no significant link between watching TV more than 3 hour with risk of overall LBP.	Self-related health and psychological distress	High	52
C. Thorbjörnsson (1999) ^c^	Nested case-control Swedish general population	484	26	Sedentary work	LBP	Interview	Sedentary work was associated with an excess risk of LBP.	Age	High	68
D. K. Shehab (2003)^c^	Cross-sectional Schoolchildren in Kuwait	400	14.4	Watching TV time.	LBP	Interview	back pain is associated with TV watching time.	-	Moderate	53
A. N. Sjolie (2004)	Cross-sectional Norwegian adolescents	88	14.7	television or computer use	LBP	Self-reported Questionnaire	LBP was associated with the use of television or computer (>15 hours/week).	Gender, distances to school and physical activity	High	26
V. ­Yip (2004)^c^	Cohort Hong Kong nurses	144	31.1	Sedentary leisure time activity and prolong setting and standing	New LBP	Face-to-face interview	leisure time sedentariness was not associated with new low back pain.	-	High	54
S. Andrusaitis (2006)^c^	Cross-sectional Brazilian truck drivers	410	40.2	Number of working hours	LBP	Questionnaire	Working hours were associated with LBP.	Weight, height, number of working hours, ethnic group, age and body mass index.	Moderate	5
V.M. Mattila (2007)	Survey; Finland Military	7040	19	No leisure-time physical activity	lifetime LBP	Visit to a physician	use of smokeless tobacco (OR 1.4) was a risk factor for LBP.	Age	High	27
P. Spyropoulos (2007)	Cross-sectional Greek office workers	648	44.5	Sitting time	Point, one year, two year, and lifetime LBP	Self-reported Questionnaire	Sitting time (>6 hours) is a significant determinant for lifetime LBP.	Gender, age, BMI, Body distance from computer screen, psychosocial factors	High	28
J. Auvinen (2008)	Cross-sectional Finland adolescents	5999	16	TV viewing, playing or working on a computer, reading books and other sitting activities	LBP	One-item question	Among girls, high amount of sitting associated with consultation or reporting LBP.	Smoking, levels of physical activity and BMI	High	29
A. Karahan (2008)	Cross-sectional Turkish hospital staff	1600	28	Standing and sitting in a working day	LBP	Questionnaire	Overall standing time in a work day was significantly associated with LBP in univariate analysis.	Occupation, gender, working year, Smoking, exercise, Perceived stress level in work environment, performing risky activities as below	High	30
B. Skoffer (2008)	Cross-sectional Danish schoolchildren	546	15.5	Standing talking during school break, hours of watching TV or video	LBP, function-limiting LBP	Self-reported Questionnaire	Homework and TV watching time were associated with LBP.	Age, gender, inactivity indicators, weight, BMI and smoking	Moderate	31
S. Ahn (2009)	Cross-sectional Korean postmenopausal women	143	59	Being inactive during leisure time	LBP	One-item question	leisure time inactivity was a risk for back pain.	health factors related to BMI, parity, osteoarthritis, BMD, drinking coffee and inactivity	High	32
F. Tissot (2009)	Population survey Canadian worker	7730	41.5	Standing at work without freedom	LBP	Standardized Nordic questionnaire	Standing at work is associated with low back pain.	-	High	33
W. Yao (2012)^c^	Case-control Chinese adolescents	1214	15.1	Prolong TV watching, computer using, and homework time	LBP	-	There was no significant association between sedentary activity and LBP.	-	High	55
N. Aggarwal (2013)^c^	Cross-sectional Indian undergraduate medical students	160	20.6	Regular watching TV, and working on PC/laptop	LBP	Questionnaire	No associations between watching television or computer use with LBP.	-	High	62
M. Mohseni Bandpei (2014)	Cross-sectional Iranian teacher	586	37.9	Standing or sitting time, computer working hours	Lifetime LBP	Oswestry LBP and disability questionnaire	Greater risk of LBP was observed in participants with prolonged sitting and standing, higher computer working hours and correcting examination.	General health, years of teaching, do exercise, pain intensifiers, sex, age, BMI, and job satisfaction	High	34
P. Mikkonen (2015)^c^	Cohort Finland Students	1625	16	working on a computer, watching television, reading books, and other sedentary activities	Chronic LBP	Self-reported or consultation-reported	No association between the sedentary behavior and LBP	-	High	66
J. Fernandes (2015)	Cross-sectional Brazilian schoolchildren	1461	12.6	Watching TV/ week	Chronic LBP	Nordic Questionnaire	Low back pain was associated with watching TV (>3 times/week and 3 hours/day)	Age, gender	High	35
G. Inoue (2015)	Cross-sectional Japanese sitting worker	1329	40	Routine standing work	LBP	RDQ	standing was not a significant risk factor for LBP.	working status, height, age, gender, BMI, smoking habit, and frequency of exercise	High	36
N. Gupta (2015)^c^	Cross-sectional Danish blue-collar workers	201	44.7	Total sitting time (hour per day)	Intensity of LBP	Standardized Nordic questionnaire	Positive association between total sitting time and high LBP intensity (OR = 1.43) was found.	Age, gender, smoking, BMI, occupational time, sitting time in the opposite domain	High	63
J. Stričević (2015)	Cross-sectional Nursing personnel in Slovenia	659	-	Working with computer or watching TV ≥ 2 h per day	LBP	Questionnaire	Work with the computer reduced the risk for LBP (OR = 0.6)	Preventive exercises, Duration of employment, Frequent manual lifting	Moderate	37
M. Dolphens (2016)	Cross-sectional Belgian adolescents	842	11.6	screen time, homework time and reading outside of school.	LBP	Questionnaire	Sedentary behaviors were not associated with LBP.	-	High	38
S. Hussain (2016)^c^	Cohort Australian adults	5058	-	Total time spent watching Television ≥ 2h/day	disability of LBP LBP intensity	Self-administered Chronic Pain Grade Questionnaire	≥ 2 hours/day TV watching was associated with greater prevalence of LBP in women	Age, smoking status, dietary index score, BMI, education,	High	65
M. Ardahan (2016)	Cross-sectional Turkish computer-using office workers	395	45	Daily working hours at computer	LB disorder	Turkish-Cornell Musculoskeletal Discomfort Questionnaire	Using a computer (> 7 hours/day) was associated with LB disorder.	-	High	39
L. Lunde (2017)^c^	Cohort Norwegian construction and healthcare workers	124	42.2	sitting and standing during work	LBP intensity	-	The duration of sitting during work and leisure time was associated with LBP intensity.	Age, gender, smoking, BMI, heavy lifting, and sitting or standing time	High	56
S. Şimşek (2017)	Cross-sectional healthcare workers (Turkey)	1682	37.9	Prolong standing & siting at work & using computer for more than 4 hours	Lifetime; Recent and previous year LBP	SNMA Questionnaire Pain level with visual analogue scale	Working for more than 4 hours (standing or sitting at desk) and using computer for more than 4 hours were associated with low back pain.	-	High	49
Y. Yabe (2017)	Cross-sectional Japanese school-aged athletes	6441	11	Video playing and TV viewing time	LBP	Self-reported questionnaire	Long video playing time/day was associated with low back pain.	Gender, age, BMI, TV-viewing time per day, and video playing time per day	High	40
S. Ganesan (2017)^c^	Cross-sectional Indian young adult	1355	24.5	Study time more than 5h/day	LBP score	Questionnaire	LBP is triggered by >5 hours studying.	-	Moderate	57
M. Balling (2017)^c^	Cohort Danish adults	76438	47.6	Total sitting time 6h/day or more	LBP	National Patient Register	No statistically significant association between total sitting time and low back pain was found.	Sex, age, smoking, BMI and physical activity at work	High	58
S. Ye (2017)^c^	Cross-sectional Chicness office workers	417	29.1	Computer use ≥8 hours/day	LBP	Oswestry Low Back Pain Disability Index	Computer use (>8 hours/day) was not associated with high LBP.	-	High	64
H. Yang (2018)	Cross-sectional USA adult population	122,337	51.5	Leisure time physical activity	Chronic LBP	Self-reported LBP	Higher prevalence of LBP among inactive people in leisure time.	Gender, age, ethnicity, socioeconomic status, and serious psychological distress	High	41
Sh. Sen Sribastav (2018)	Cross-sectional; Patient with LBP (Chi na)	1046	37.2	Long time driving	Non-specific LBP and pain level	Self-assessment question­naire	Smoking, long driving time, and higher BMI were associated with LBP pain.	Gender, age, BMI, smoking habits, duration of driving or riding, drinking habits	High	42
S. Park (2018)	Cross-sectional General Korean population	5364	65.4	sitting time	Chronic LBP	Self-reported LBP	Sitting time more than 7 hours/day was notably associated with LBP.	Age, socioeconomic factors, sex, BMI, smoking history, alcohol consumption, and physical activity	High	43
M. Korshøj (2018)^c^	Cross-sectional	704	45	Sitting at work	Intensity of LBP	Nordic Questionnaire	No significant associations were found between total duration and temporal patterns of sitting with LBP.	Interaction between sitting and BMI	High	13
A. Citko (2018)	Survey Poland medical personnel	609	41	Sedentary lifestyle	Recurrent and chronic LBP	Nordic musculoskeletal questionnaire	Sedentary lifestyle associated with a 3.5-fold increase in the incidence of LBP.	-	High	19
S. Çelik (2018)^c^	Cross-sectional Office worker	528	38.6	Time spent standing and continuously sitting in workplace	LBP	Questionnaire	There is no significant association time spent standing and sitting with risk of LBP.	-	High	59
R. Shiri (2018)^c^	Cohort Finnish population	3505	-	Sitting hours/day	LBP	Questionnaire	No significant associations between sitting time/day and LBP for more than 7 or 30 days.	Age, sex	High	67
S. Kulandaivelan (2018)	Cross-sectional HISAR urban population	1540	48.2	Long sitting or watching TV time (> 2h/day)	LBP	Modified Nordic musculoskeletal questionnaire	No statistically significant association between long sitting and sleeping time and LBP	-	High	44
C. Tavares (2018)^c^	Cross-sectional Brazilian medical students	629	23	Sitting hours per day	LBP	Questionnaire	The number of sitting hours was not associated with LBP.	-	High	60
Q. Zhang (2019)	Cross-sectional Emergency ambulance workers (driver). (China)	1560 (543 drivers)	38.4	Sitting time	Chronic LBP	Nordic Musculoskeletal Questionnaire	BMI and sitting time were associated with chronic LBP study sample.	Age, BMI, sex, Psychosocial factors	High	45
B. Minghelli (2019)	Cross-sectional Portuguese students	304	13.7	Sedentary habits (Watching television, Mobile phones use, Console/computer games)	Lifetime and 6- and 12-month LBP	Self-Questionnaire	Mobile use more than 10 hours/week is associated with LBP in adolescents.	Sex, sedentary habits, physical activity	High	46
T. Bento (2019)	Cross-sectional Brazilian students	1628	15	Daily use time of TV/day, cell phone, tablet more than 3 h	Chronic LBP	Nordic Questionnaire	Daily TV use, cell phone use and tablet use (>3 hours) were significantly associated with LBP.	Sex, TV watching hours, daily use time of cell-phone and tablet	High	47
H. Ayed (2019)	Cross-sectional Tunisia schoolchildren	1221	15.6	Watching TV more than 12h/week and playing video game for more than 2h/week	LBP	Nordic Questionnaire	Playing videogames (≥2 hours/day), and watching TV (≥12 hours/week) were significant risk factors for LBP.	-	High	20
F. Hanna (2019)	Cross-sectional Qatari University Employees	479	-	Prolong sitting hour	LBP	ALBPSQ	Too much sitting was significantly associated with LBP or UBP	Age, gender, and profession.	High	48
D. Schwertner (2019)^c^	Cross-sectional Brazilian young	330	16	TV watching time and computer use	LBP	Oliveira Questionnaire on Low Back Pain in Youths	No association of LBP with sedentary lifestyle was found.	Sex, age and BMI	High	61
C. Bontrup (2019)^c^	Cross-sectional Swedish call-center employees	70	43	Occupational sitting habits	LBP	CPG questionnaire and BPI	Small association between general sitting behavior and chronic LBP was found.	-	High	1

Abbreviations: STROBE; STrengthening the Reporting of OBservational studies in Epidemiology; BMI, body mass index; BMD, bone mass density; LBP, low back pain; RDQ, Roland-Morris Disability Questionnaire; CPG, Chronic Pain Grade; BPI, Brief Pain Inventory; ALBPSQ, Acute Low Back Pain Screening Questionnaire. ^a^Arranged chronologically. ^b^ Mean STROBE score ≥ 16.5 (from two reviewers) was considered as high quality and 11 to 16.5 was considered as moderate quality. ^c^ Not included in metanalyses.

### Assessment of study quality

Mean STROBE scores from two reviewers revealed 42 studies conducted in high quality and 7 studies in a moderate quality. We used all these studies in data synthesis or meta-analysis since, concerning moderate quality studies, none of those had small sample sizes or inaccurate estimates. Besides, in the sensitivity analyses, all of the studies were excluded, and the effect sizes were estimated again to ensure the accuracy of data. The quality of each selected study is presented in Table 1. More details about the assessment of study qualities are presented in Appendix 2.

### Main findings of the meta-analysis

**Figure 2:**
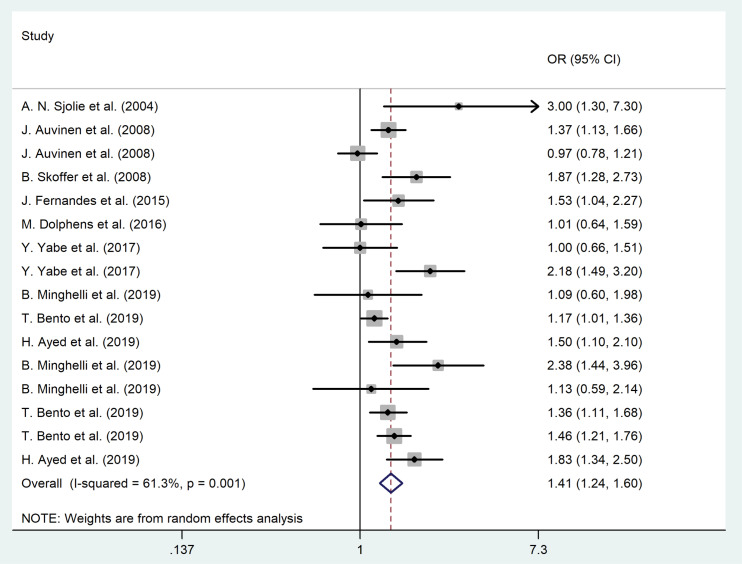
The association between sedentary lifestyle and LBP among children and adolescents.

The forest plot for the association between sedentary behaviors and LBP among children and adolescents is shown in Figure 2. The pooled odds ratio (based on cross-sectional studies) illustrated that sedentary lifestyle was a remarkable risk factor for LBP among children and adolescents (OR = 1.41, 95% CI = 1.24–1.60, *P* = 0.002; I^2^ = 66.5%, *P* = 0.001). No evidence of publication bias was noted (for all studies, *P* value of Egger’s test = 0.40, and *P* value of Begg’s tests = 0.19).

**Figure 3:**
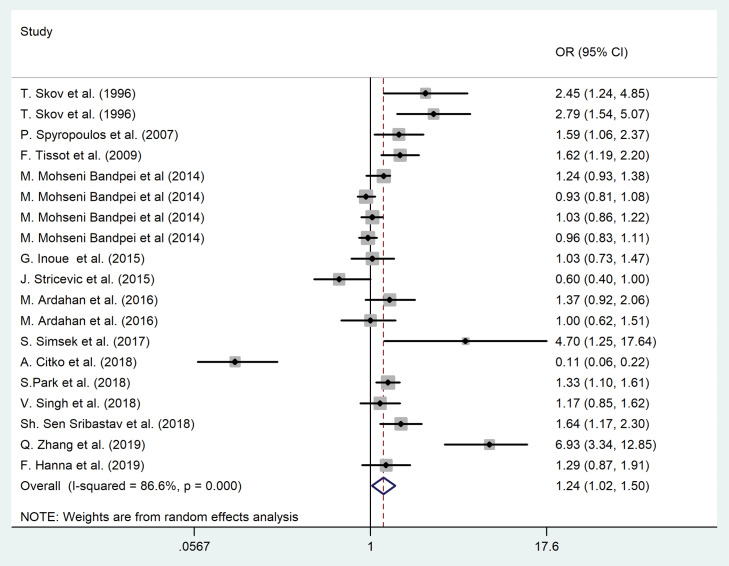
The association between sedentary lifestyle and LBP among adult population.

The forest plot for the correlation of sedentary behaviors and LBP among the adult population is shown in Figure 3. The pooled odds ratio (based on cross-sectional studies) illustrated that the sedentary lifestyle was a considerable risk factor for LBP among the adult population (OR = 1.24, 95% CI = 1.02-1.50, *P* <0.001; I^2^ = 84.8%, *P* < 0.001). No evidence of publication bias was noted (for all studies, *P* value of Egger’s test = 0.91, and *P* value of Begg’s tests = 0.08).

### Subgroup meta-analysis according to the type of sedentary behaviors 

**Figure 4:**
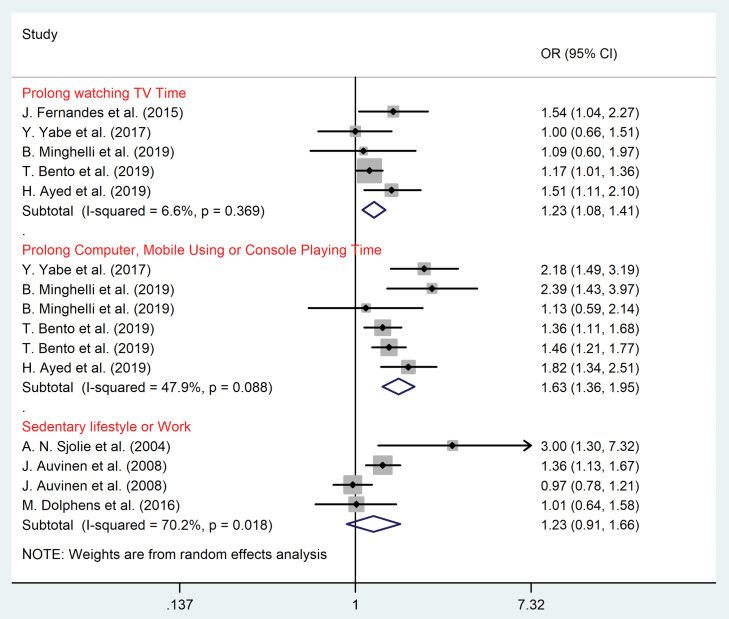
Subgroup analysis according to sedentary lifestyle among children and adolescents.

**Figure 5:**
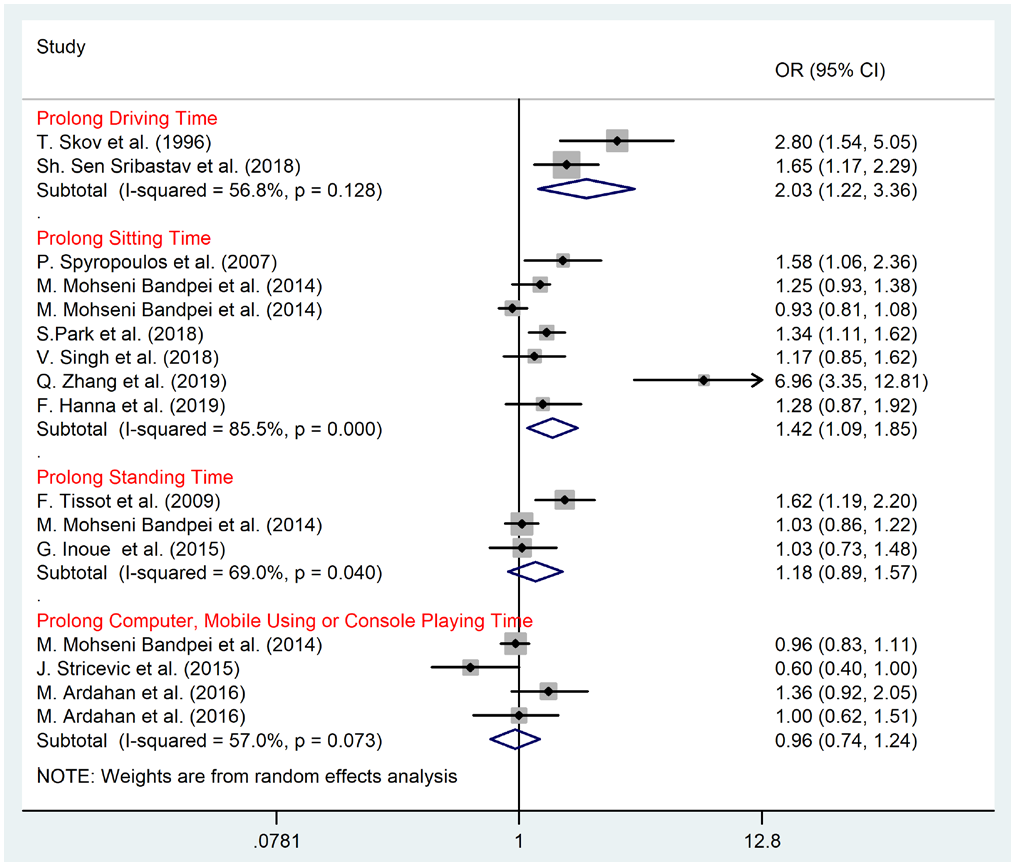
Subgroup analysis according to sedentary lifestyle among adult population.

Results of subgroup analysis based on the type of sedentary behaviors among children/adolescents and adult populations are shown in Figure 4 and Figure 5, respectively. Among children and adolescents prolonged watching TV (OR = 1.23, 95% CI = 1.08–1.41, *P* = 0.003; I^2^ = 6.6%, *P* = 0.37), computer/mobile using and console playing time (OR = 1.63, 95% CI = 1.36–1.95, *P* = 0.001; I^2^ = 47.9%, *P* = 0.09) were significant risk factors for LBP (*P* value < 0.05) (Figure 4).

Among adult population prolonged sitting time (OR = 1.42, 95% CI = 1.09–1.85, *P* = 0.03; I^2^ = 85.5%, *P* < 0.001), and driving time (OR = 2.03, 95% CI = 1.22–3.36, *P* <0.001; I^2^ = 56.8%, *P* = 0.13) were the significant risk factors for LBP (Figure 5).

### Subgroup meta-analysis according to occupation type

**Figure 6:**
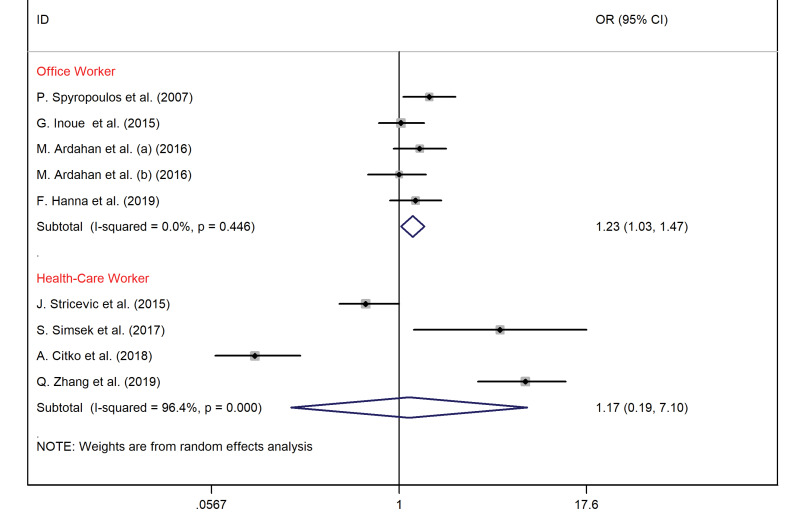
The association between the sedentary lifestyle and LBP according to occupation type.

Among office workers, sedentary lifestyle was an essential risk for LBP (OR = 1.23, 95% CI = 1.03–1.47, I^2^ = 0%) (Figure 6).

### Body mass index and smoking status

**Figure 7:**
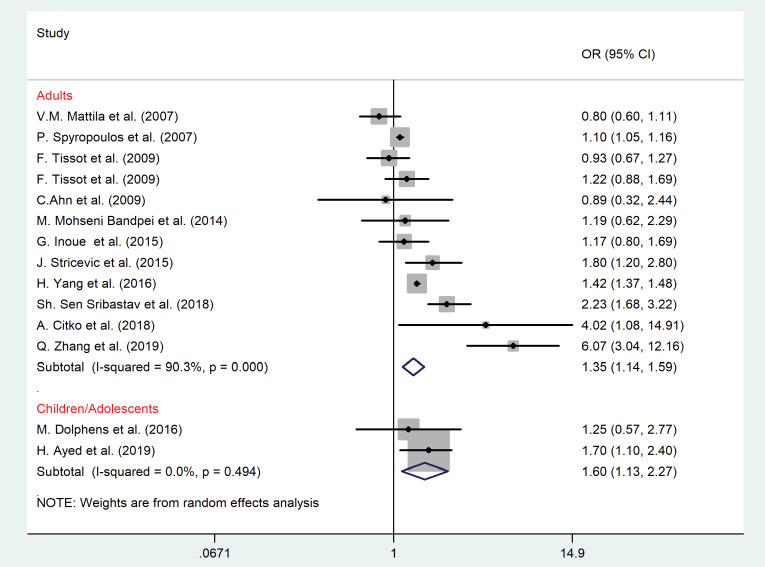
The association between Body Mass Index (BMI) and LBP according to the age group.

**Figure 8:**
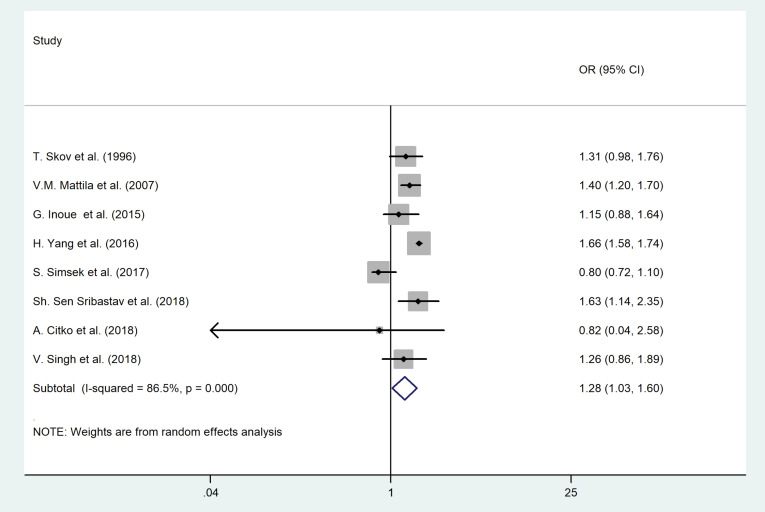
The association between smoking and LBP among adult population.

As shown in Figures 7 and 8, overweight or obesity (OR = 1.35, 95% CI = 1.14–1.59, *P* = 0.02; I^2^ = 90.3%, *P* < 0.001) and smoking (OR = 1.28, 95% CI = 1.03–1.60, *P* = 0.01; I^2^ = 86.5%, *P* < 0.001) were the significant risk factors for LBP among adult population. In children, excess wight (OR = 1.60, 95% CI = 1.13–2.27, *P* = 0.021; I^2^ = 0.00, *P* = 0.49) was associated with LBP as well.

No evidence of publication bias for BMI was found (*P* value of Egger’s test = 0.41, and *P* value of Begg’s tests = 0.68).

We found publication bias for smoking (*P* value of Egger’s test = 0.71, and *P* value of Begg’s tests = 0.03). Therefore, we conducted the Trim and Fill method to explore the effect of publication bias on the meta-analysis results. However, no significant change in the pooled OR was noted.

### Leisure time inactivity

**Figure 9:**
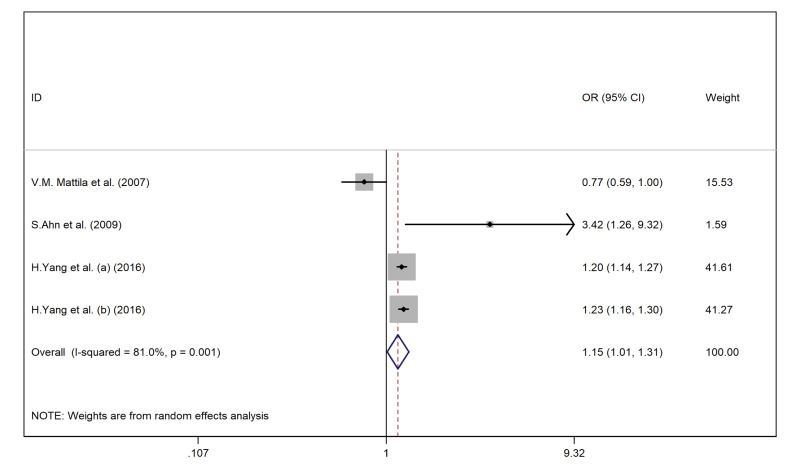
The association between leisure-time inactivity and LBP.

As shown in Figure 9, leisure time inactivity was an essential risk factor for LBP (OR = 1.28, 95% CI = 0.92–1.77, I^2^ = 81%).

### Sensitivity analysis

We found no significant changes between the before-after sensitivity pooled OR for the association between sedentary lifestyle and LBP among children and adolescents. However, results showed a remarkable effect between before-after sensitivity pooled OR for the correlation between the sedentary lifestyle and LBP among adult population after excluding Zhang et al^45^ study (OR = 1.16, 95% CI = 0.99–1.36).

Also, results showed significant changes between the before-after sensitivity pooled OR for the association between smoking and LBP among the adult population after excluding Mattila et al^27^ study and Sribastav et al^42^ study (OR = 1.26, 95% CI = 0.97–1.56).

Besides, no remarkable changes between the before-after sensitivity pooled OR for the association between the sedentary lifestyle and LBP among healthcare workers and office workers were noted.

### Overview of studies not included in the meta-analysis

Sedentary behavior at work with non-neutral posture correlates with LBP among workers.^50^ Bending postures but not sedentary leisure time itself have been proposed to be associated with new LBP in nurses.^54^ However, three studies stated no association between sedentary habits and LBP.^55,61,62^

Sitting behavior was associated with chronic LBP and functional disfunction among 70 call center employees.^1^ Among a population of truck drivers, the only factor correlated to LBP was the number of working hours.^5^ Also, the daily number of studying hours (>5 hours) precipitated the LBP in young adults.^57^ On the other hand, sitting time was not considered a risk factor for LBP in 3 studies.^58-60^

LBP was reported more in school children playing videogames >2 hours/day and not for television watchers in the Gunzburg et al study.^51^ Similar to this finding, a cohort study by Croft et al reported that watching television > 3 hours/day did not enhanced the risk of recurrent LBP in the UK general population.^52^ Similarly, in a cohort study among Finnish children, the sedentary class boys (derived from latent class analysis) did not presented with increased risk for reporting LBP or consultation for LBP.^66^ More conflicting results have been reported in Shehab et al study in which the LBP correlated with female gender and TV watching time in children and adolescents.^53^

In Hussain et al. study, TV watching time in women was associated with greater LBP disability. The authors suggested that targeting the time spent TV watching would be effective in reducing LBP disability in adults at the level of community.^65^ A retrospective study revealed that sedentary work was associated with LBP in both genders after a 24 year period.^68^ Also, in Shiri et al study, lifestyle including abdominal obesity and smoking increased the risk of LBP. Reduced risk of LBP was obtained via walking and cycling to work (OR = 0.75).^67^

Regarding the intensity of LBP, Gupta et al showed a notable association between total sitting time and high LBP intensity among 201 participants (OR = 1.43).^63^ The duration of sitting time both in work and leisure time was associated with LBP intensity in another study.^56^ Such a relationship was investigated in Ye et al study among 417 office workers. In contrast, in this study computer use ≥ 8 hours/day was not associated with high intensity LBP.^64^ In the Korshøj et al study, the sitting pattern was not correlated with the intensity of LBP.^13^ Moreover, in Hussain et al. cohort study on 5058 individuals, no significant associations between < 2.5 hours/week physical activity and ≥ 2 hours/day TV watching, with LBP intensity at follow-up were reported.^65^ Thus, further research is necessary to better elucidate the effect of sedentariness on the risk of high-intensity LBP.

### Coffee drinking and LBP

In a survey, Citko et al showed that coffee drinking, 6 cups per day or more, increased the risk of non-specific LBP recurrence by 16 times compared to smaller amounts in medical personnel.^19^ Also, a survey of 134 postmenopausal women reported a significant association of drinking coffee (yes/no) with LBP (OR = 3.1).^32^ However, in the Aggarwal study, regular or occasional coffee intake was not associated with LBP among undergraduate students.^62^ The absence of association was found in the Ganesan et al study as well.^57^ Abovementioned studies were all cross-sectional in their design. The effect of coffee on back pain may be dose-dependent or through indirect mechanisms such as via affecting bone health.^32^ To better clarify this issue, further exploration is necessary, with a standard measurement of coffee/caffeine intake, especially in longitudinal research.

## Discussion

Our study explored the association of sedentary behavior and LBP. The results demonstrate the role of sedentary behavior as a risk factor for the increased incidence of LBP, both in adults and children (OR = 1.24 and 1.41, respectively).

A similar systematic review to ours, published in 2009, was performed on 15 observational studies up to 2006 and revealed that there was no correlation between sedentary behavior both in work or leisure time with LBP.^12^ However, given that more articles have been published in recent years, we were able to obtain the pooled OR for the abovementioned association for adults and children separately. In addition, in the previous review, only prolonged sitting was considered as sedentary behavior, whereas we conducted subgroup meta-analyses according to the type of sedentary behaviors and occupation type.

The time spent in sedentary lifestyle has become a significant health concern. The sedentary behavior prevalence is high, even in developed countries.^69^ One study showed that children spend 51.4% of their working time in sedentary lifestyle. These common behaviors may be established in childhood and track through later life.^70^ Sedentary behavior is linked to various musculoskeletal pain conditions.^71^

Despite the controversies observed in included articles in our systematic review, the pooled effects sizes obtained from meta-analysis of other studies revealed that prolonged sitting time and prolonged driving time are significant risk factors of LBP among adults (OR = 1.42, 2.03 respectively). However, prolonged screen time and standing time were not associated with LBP in adults. Also, among children, prolonged TV watching (OR = 1.23), and computer/mobile using or console playing time (OR = 1.63) were associated with LBP. A meta-analysis study indicated that excess weight is a risk factor for LBP in both genders.^72^ Another study revealed that smokers have a higher incidence of LBP compared to nonsmokers; these associations were fairly modest (OR = 1.32 for former and OR = 1.31 for the current smokers). Of note, the association between current smoking and LBP was more remarkable in adolescents than in adults (OR = 1.82 vs. 1.16).^73^ Similarly, we found that increased body mass index in adults and children and smoking in adults, are risk factors for LBP (OR = 1.35, 1.60 and 1.28 respectively), in whom the sedentary behavior has been investigated. These finding suggest that smoking and body mass index interact with sitting and LBP.^13^ In addition, to avoid heterogeneity, we identified two main occupation categories as healthcare workers and office workers among the included studies. We found that sedentary behavior is a risk for LBP in office workers (OR = 1.23). In previous research, prolonged sitting and computer use were contributed to LBP in office workers.^48^

As for underlying pathways, decreased level of water supply to the vertebral disc, which in turn leads to degenerative changes and disk herniations, reduced strength and muscular power, and developing hyperlordosis are some proposed pathophysiological mechanisms for sedentary behavior contributing to LBP.^19^ Specifically, prolonged sitting is contributed to decreased postural change, as well as muscle strength and disk degenerations.^13^ Obesity or overweight causes overload on the spinal tissues and contributes to disk herniation and LBP. On the other hand, obesity is associated with other disorders such as diabetes and hyperlipidemia that are also correlated to LBP by different mechanisms.^74^ Smoking can alter the blood supply of vertebral disks via the processes of vasoconstriction and atherosclerosis. Impaired perfusion of vertebral structures leads to degenerative changes and LBP. Besides, smoking is a risk factor of osteoporosis or is a behavior seen only the people with massive physical works; thus, it has direct and indirect effects on the LBP.^73^ Moreover, coffee consumption is proposed to be associated with flushing magnesium from the body and increased painful contractions of paraspinal muscles.^75^

The data heterogeneity of included studies in our review can be explained in part by variations in study designs, study population, sample sizes, occupation type, gender, race, and age range. However, beyond those, some factors seem to be more important, as follows.

First, the definition of LBP and its measurement scales were considerably different in the studies. For instance, experiencing LBP during the current week for at least 48 hours via the Roland-Morris Disability Questionnaire was measured in the Inoue et al. study.^36^ In the Ben Ayed et al study, however, participants were asked about discomfort and pain in the low back area during the prior month.^20^ Some authors, though, defined recurrent LBP as pain episodes of at least three times in the last 12 months and chronic LBP as the pain persisted for at least 12 weeks based on the Nordic Musculoskeletal Questionnaire.^19^ Many studies, however, did not differentiate chronic LBP from acute LBP.

Second, sedentary behavior has diverse definitions and types in various studies. While some authors explored the association of sitting time merely with LBP,^28^ some others have turned their attention to the screen time or a combination of both.^49^ In some other studies, sedentary habits were not categorized into any different types.^68^ In addition, the common measurement tool for sedentary behavior is subjective self-reported questionnaires, which, in turn, are prone to information bias from participants. However, a few studies used objective-based tools such as an accelerometer or textile pressure mat to estimate the sitting time.^1,63^ Thus, to make accurate estimations, we performed subgroup analyses for specified sedentary behavior in the papers, both for adults and children separately. For future research, focusing on objective-based measurement of sedentary behaviors is highly suggested.

The third is that LBP, as a complex multifactorial disease, is affected by psychological conditions and the tasks performed in non-sitting positions at work or leisure time. Therefore, just a part of the variation in LBP is because of sedentary-related risk factors.^33,48^ Thus, the variety in the combination of these factors in different participants seems to be accounted for the data heterogeneity.

### Strengths of the study

We applied different statistical methods to obtain the desired associations for adults and children separately, as the risk factors of LBP may be different in these age groups. We found new data and demonstrated significant but moderate associations between different sedentary behaviors and LBP. Regarding the large number of studies conducted in this field (which were retained in the meta-analysis), the results can be well generalized to different communities.

### Health implications

A recent meta-analysis evaluating the lifestyle interventions to reduce sedentary behavior among five categories of population with a clinical condition (including musculoskeletal conditions) demonstrated that after multicomponent interventions, individuals with different medical conditions successfully reduced their sedentary behavior (by 64 minutes/day). The interventions consisted of the use of technologies, social facilitation, motivational counselling and self-monitoring.^76^ As LBP is a complex disorder, health education to reduce the prevalence or occurrence of LBP should be address the risk factors as much as possible including sedentary behavior.

## Conclusion

In brief, according to our meta-analysis, sufficient evidence exists from recent studies that indicate the association of different types of sedentary behavior with the occurrence or recurrence of LBP both in adults and children. Given the increasing trend of sedentary behavior worldwide, especially in the era of the COVID-19 pandemic, meticulous and robust preventive strategies are suggested to be applied to avoid the establishment of sedentariness early in childhood and to prevent its’ musculoskeletal consequences such as LBP.

## Acknowledgments

We want to thank our colleagues in Isfahan University of medical Sciences who helped us working on this project.

## Funding

This study was funded and supported by Isfahan University of Medical Sciences, Isfahan, Iran.

## Competing interests

None to declare.

## Ethical approval

The protocol of the current review has been qualified in the Isfahan University of Medical Sciences, Isfahan, Iran (code: 199298) and has been approved in the National regulatory ethics committee (IR.MUI.MED.REC.1399.507). The study protocol and its details have been registered in the international prospective register of systematic reviews, PROSPERO with identification code: CRD42020187175.

## Authors’ contributions

SBM contributed to the conception of the work, data search, screening of records, study quality assessment, data extraction, manuscript preparation, manuscript revision, final approval of the manuscript, and agreed to be accountable for all aspects of the work. RR contributed to the screening of records, study quality assessment, data extraction, statistical analysis, interpretation of data, manuscript preparation, manuscript revision, final approval of the manuscript, and agreed to be accountable for all aspects of the work. BV contributed to study quality assessment, manuscript preparation, manuscript revision, final approval of the manuscript, and agreed to be accountable for all aspects of the work. RK contributed to the conception of the work, manuscript preparation, manuscript revision, final approval of the manuscript, and agreed to be accountable for all aspects of the work. All authors approved the final version of manuscript and took the responsibility for all aspects of the work.

## Appendix 1

**
Search string for PubMed
**

((((«Sedentary Behavior»[Mesh] OR «Screen Time»[Mesh]) OR «Coffee»[Mesh]) OR «Tea»[Mesh]) OR ((«tea»[MeSH Terms] OR «tea»[All Fields]) OR («coffee»[MeSH Terms] OR «coffee»[All Fields]) OR («caffeine»[MeSH Terms] OR «caffeine»[All Fields]) OR «sedentary lifestyle»[All Fields] OR «physical inactivity»[All Fields] OR «sedentary behavior*»[All Fields] OR «screen time»[All Fields] OR «sitting time»[All Fields] OR sedentary[All Fields] OR «watching TV»[All Fields] OR «playing video game*»[All Fields] OR ((«work»[MeSH Terms] OR «work»[All Fields] OR «working»[All Fields]) AND («computers»[MeSH Terms] OR «computers»[All Fields] OR «computer»[All Fields])))) AND («Low Back Pain»[Mesh] OR («low back pain»[All Fields] OR «back pain»[All Fields] OR «spinal pain»[All Fields] OR «spine pain»[All Fields] OR («low back pain»[MeSH Terms] OR («low»[All Fields] AND «back»[All Fields] AND «pain»[All Fields]) OR «low back pain»[All Fields] OR «lumbago»[All Fields]) OR («back pain»[MeSH Terms] OR («back»[All Fields] AND «pain»[All Fields]) OR «back pain»[All Fields] OR «backache»[All Fields]) OR «lumbar spondylosis»[All Fields] OR «postural low back pain»[All Fields] OR «mechanical low back pain»[All Fields]))

548 Records [with these filters: full text, humans, English]

**
Search string for Web of Science
**

TOPIC: (tea OR coffee OR caffeine OR “sedentary lifestyle” OR “physical inactivity” OR “sedentary behavior*” OR “screen time” OR “sitting time” OR sedentary OR “watching TV” OR “playing video game*” OR “working on a computer”) AND TOPIC: (“low back pain” OR “back pain” OR “spinal pain” OR “spine pain” OR lumbago OR backache OR “lumbar spondylosis” OR “postural low back pain” OR “mechanical low back pain”)

492 Records [with these filters: article, English]

**
Search string for Scopus
**

( ALL ( tea OR coffee OR caffeine OR «sedentary lifestyle» OR «physical inactivity» OR «sedentary behavior*» OR «screen time» OR «sitting time» OR sedentary OR «watching TV» OR «playing video game*» OR «working on a computer» ) AND TITLE-ABS-KEY ( «low back pain» OR «back pain» OR «spinal pain» OR «spine pain» OR lumbago OR backache OR «lumbar spondylosis» OR «postural low back pain» OR «mechanical low back pain» ) ) AND ( LIMIT-TO ( DOCTYPE, «ar» ) ) AND ( LIMIT-TO ( LANGUAGE, «English» ) ) AND ( LIMIT-TO ( SRCTYPE, «j» ) )

1744 Records [with these filters: article, journals, English]

**
Search string for Embase
**

(tea OR coffee OR caffeine OR ‹sedentary lifestyle› OR ‹physical inactivity› OR ‹sedentary behavior*› OR ‹screen time› OR ‹sitting time› OR sedentary OR ‹watching tv› OR ‹playing video game*› OR ‹working on a computer›) AND (‹low back pain› OR ‹back pain› OR ‹spinal pain› OR ‹spine pain› OR lumbago OR backache OR ‹lumbar spondylosis› OR ‹postural low back pain› OR ‹mechanical low back pain›)

622 Records [with this filter: article]

## Appendix 2

**Appendix 2:** Quality assessment of included articles using the STROBE checklist

**Author;** **(year)**	**STROBE score (Reviewer 1: S.B.M)**	**STROBE score (Reviewer 2: B.V)**	**Mean STROBE score**	**Study quality** ^1^
A. Burdorf (1993)	16	16	16	**Moderate**
T. Skov (1996)	16	16	16	**Moderate**
R. Gunzburg (1999)	17	16	16.5	High
P. R.Craft (1999)	21	18	19.5	High
C. Thorbjornsson (1999)	19	17	18	High
D. K. Shehab (2003)	15	12	13.5	**Moderate**
A. N.Sjolie (2004)	17	16	16.5	High
V. ­Yip (2004)	18	19	18.5	High
S. Andrusaitis (2006)	17	14	15.5	**Moderate**
V.M. Mattila (2007)	19	17	18	High
P. Spyropoulos (2007)	19	19	19	High
J. Auvinen (2008)	21	19	20	High
A. Karahan (2008)	20	20	20	High
B. Skoffer (2008)	17	15	16	**Moderate**
S. Ahn (2009)	19	20	19.5	High
F.Tissot (2009)	19	20	19.5	High
W. Yao (2012)	20	19	19.5	High
N. Aggarwal (2013)	19	18	18.5	High
M. Mohseni Bandpei (2014)	19	18	18.5	High
P.Mikkonen (2015)	20	17	18.5	High
J. Fernandes (2015)	18	15	16.5	High
G. Inoue (2015)	17	17	17	High
N. Gupta (2015)	22	20	21	High
J. Stricevic (2015)	15	14	14.5	**Moderate**
M. Dolphens (2016)	19	18	18.5	High
S. Hussain (2016)	21	19	20	High
M. Ardahan (2016)	19	15	17	High
L. Lunde (2017)	20	18	19	High
S. ŞIMŞEK (2017)	17	19	18	High
Y. Yabe (2017)	21	18	19.5	High
S. Ganesan (2017)	15	17	16	**Moderate**
M. Balling (2017)	21	20	20.5	High
S.Ye (2017)	17	18	17.5	High
H. Yang (2016)	17	18	17.5	High
Sh. Sen Sribastav (2018)	18	19	18.5	High
S.Park (2018)	21	17	19	High
M. Korshøj (2018)	21	20	20.5	High
A. Citko (2018)	18	17	17.5	High
S. Celik (2018)	16	18	17	High
R. Shiri (2018)	21	18	19.5	High
S. Kulandaivelan (2018)	17	17	17	High
C. Tavares (2018)	18	16	17	High
Q. Zhang (2019)	18	18	18	High
B. Minghelli (2019)	15	18	16.5	High
T. Bento (2019)	20	20	20	High
H. Ayed (2019)	20	20	20	High
F. Hanna (2019)	18	19	18.5	High
D. Schwertner (2019)	18	18	18	High
C. Bontrup (2019)	16	18	17	High

STROBE: STrengthening the Reporting of OBservational studies in Epidemiology. 1: Mean STROBE score ≥ 16.5 (from two reviewers) was considered as high quality and 11 to 16.5 was considered as moderate quality.
